# Efficacy of Azithromycin in Preventing Pulmonary Exacerbations Among Patients With Bronchiectasis: A Systematic Review and Meta-Analysis

**DOI:** 10.7759/cureus.101574

**Published:** 2026-01-14

**Authors:** Renzxymon L Dela Cruz, Francis Adrian D Que, Louise Nicole C Bacud, Minette Claire O Rosario

**Affiliations:** 1 Department of Medicine, St. Luke's Medical Center, Quezon City, PHL; 2 Department of Infectious Diseases, St. Luke's Medical Center, Quezon City, PHL

**Keywords:** adverse events, azithromycin, bronchiectasis, meta-analysis, pulmonary exacerbations

## Abstract

Bronchiectasis is a debilitating chronic respiratory condition characterized by a vicious cycle of infection, inflammation, and airway destruction, leading to frequent pulmonary exacerbations. While long-term macrolide therapy, particularly azithromycin, has been proposed for its antibiotic and anti-inflammatory effects, previous systematic reviews have reported inconsistent findings or failed to include the most recent randomized controlled trials (RCTs). This study aimed to provide a comprehensive, updated meta-analysis of the efficacy and safety of long-term azithromycin in reducing pulmonary exacerbations. We systematically searched PubMed, ClinicalTrials.gov, and the Cochrane Library through December 2025, identifying six RCTs involving 562 patients. The analysis revealed that long-term azithromycin prophylaxis significantly reduced the rate of pulmonary exacerbations compared to placebo, with a pooled standardized mean difference (SMD) of -0.63 (95% CI, -0.90 to -0.35; p < 0.00001). Subgroup analysis demonstrated consistent benefits in both adult (SMD -1.03) and pediatric (SMD -1.52) populations (p = 0.10 for subgroup difference), despite moderate heterogeneity (I² = 58%). While treatment did not yield statistically significant improvements in forced expiratory volume in 1 second (FEV1) predicted (p = 0.08) or St. George’s Respiratory Questionnaire (SGRQ) scores (p = 0.81), the incidence of adverse events was comparable to placebo (relative risk (RR) 1.24; 95% CI, 0.95 to 1.61; p = 0.11). These findings indicate that azithromycin is a safe and effective intervention for preventing exacerbations in both children and adults and should be considered in the management of patients with frequent respiratory events.

## Introduction and background

Bronchiectasis is a chronic respiratory disease characterized by cough, purulent sputum production, dyspnea, chest pain, and recurrent chest infections [1]. Bronchiectasis is marked by a vicious cycle of persistent bacterial infection and excessive neutrophilic inflammation, leading to impairment of airway defense mechanisms and manifesting as a debilitating respiratory condition that affects people of all ages [1]. Long-term macrolide therapy, mainly azithromycin, has been a cornerstone in managing frequently exacerbating bronchiectasis in patients for over a decade [2]. However, the exact magnitude of benefit, particularly across different age groups, and the overall balance of risk (such as antibiotic resistance and QTc prolongation) versus benefit, remain contentious [3]. Previous meta-analyses have often yielded inconsistent results for the primary outcome and have been limited by the inclusion of fewer randomized controlled trials (RCTs) and by missing data from high-quality pediatric and recent adult trials, such as the studies by Stick in 2022 and Vicendese in 2023 [4,5]. The updated meta-analysis has dual significance: first, by including two of the most recently published and largest RCTs, it provides the most up-to-date and best evidence for the overall efficacy of azithromycin; second, the detailed subgroup analysis allows a better evaluation of efficacy in pediatric versus adult populations. This update thus addresses a critical question for clinical guidelines, which currently lack high-certainty evidence for children [6]. A synthesis as updated and comprehensive as this paper is imperative for informing clinical practice guidelines, patient-physician shared decision-making, and prioritizing high-yield areas for future research at a time when monitoring antibiotic resistance is critical. The objective of this meta-analysis is to assess the efficacy of azithromycin treatment versus placebo in preventing pulmonary exacerbations in patients diagnosed with bronchiectasis. Secondary objectives included the following: (1) impact of azithromycin on lung function, specifically the forced expiratory volume in 1 second (FEV1) percent predicted; (2) St. George’s Respiratory Questionnaire (SGRQ) total score to evaluate changes in health-related quality of life; and (3) safety profile and tolerability of long-term azithromycin therapy.

## Review

Methods

This review is reported in accordance with the Preferred Reporting Items for Systematic Reviews and Meta-Analyses (PRISMA) 2020 checklist. It was registered at the International Prospective Register of Systematic Reviews (PROSPERO) database (CRD420251004942).

Literature Search

We have systematically searched several databases, including PubMed, ClinicalTrials.gov, and the Cochrane Library, from inception to December 3, 2025, using the following keyword: “azithromycin [MESH].” AND “bronchiectasis [MESH].” We applied the randomized controlled trials filter. No language filters were applied during the initial search phase to ensure maximal sensitivity. The reference lists of retrieved studies and relevant reviews were also hand-searched, and the process described above was repeated to include additional eligible studies.

Inclusion and Exclusion Criteria

The inclusion criteria were presented as follows: (1) the study design was an RCT, (2) patients were diagnosed with bronchiectasis, and (3) intervention treatments were azithromycin versus placebo. Articles excluded are those with: (1) non-randomized or observational study designs; (2) studies not published as full-text articles; (3) studies published in languages other than English; and (4) studies that did not report the primary outcome of interest (number of pulmonary exacerbations).

Selection of Studies

Two authors independently screened and reviewed the abstracts and articles for inclusion. Articles were selected based on the inclusion criteria, and the decision to include the article was made through consensus. Disagreements during the review process were resolved by discussion with a third reviewer and by consensus.

Data Extraction and Outcome Measures

Baseline information was extracted from the original studies, including the first author, number of patients, age, sex, FEV1, and detailed methods for the two groups. Data were extracted independently by two investigators, and discrepancies were resolved by consensus. The primary outcome was the rate of pulmonary exacerbations. Based on conventional clinical criteria, an exacerbation was considered to be an acute exacerbation of respiratory symptoms (cough, sputum volume, or purulence) that requires systemic antibacterial therapy [1, 2]. The effect measure used for the outcome was the standardized mean difference (SMD) with 95% CI for the purpose of combining the disparate expressions of the primary outcome in the included studies as mean values or rates.

Secondary outcomes were lung function, health-related quality of life, and safety. Changes from baseline to the end of the treatment period in FEV1 % predicted were used as a measurement for the change in lung function, and the SMD was used as the size of the treatment effect. Health-related quality of life was assessed by using the total score of SGRQ (scale 0 to 100), with lower scores representing greater health-related quality of life, and the SMD for the overall comparison. Lastly, overall adverse events were used to assess safety and tolerability, and the risk ratio (RR) and the 95% CI were used for comparison between the azithromycin group and the placebo group.

Risk of Bias Assessment

We used the Cochrane Risk of Bias (RoB) 2 tool to conduct the quality assessment of each article. Each article was critically appraised for each domain of bias, including bias from the randomization process, bias due to deviations from intended interventions, bias due to missing outcome data, bias in the measurement of the outcome, and bias in the selection of the reported result. These domains were graded as high, low, or with some concern, and discrepancies were then resolved through constructive discussion and reaching a consensus.

Statistical Analysis

The meta-analysis was performed using Review Manager (RevMan) version 9.17.0 (The Cochrane Collaboration, London, UK). For the primary outcome of pulmonary exacerbations and the safety analysis of adverse events, we utilized a random-effects model to account for anticipated clinical heterogeneity between studies. However, for the secondary continuous outcomes of lung function (FEV1) and health-related quality of life (SGRQ), a fixed-effects model was employed. For continuous outcomes, SMD with 95% CIs was calculated. For dichotomous outcomes (e.g., adverse events), RR with 95% CIs was estimated. Statistical heterogeneity was assessed using the I² statistic and the chi-square test. A pre-specified subgroup analysis was performed based on age (adults vs. children). Sensitivity analysis was conducted by omitting one study at a time to assess the robustness of the primary findings. Publication bias was visually assessed using funnel plots for the primary outcome. A two-sided p-value < 0.05 was considered statistically significant.

Results

**Figure 1:**
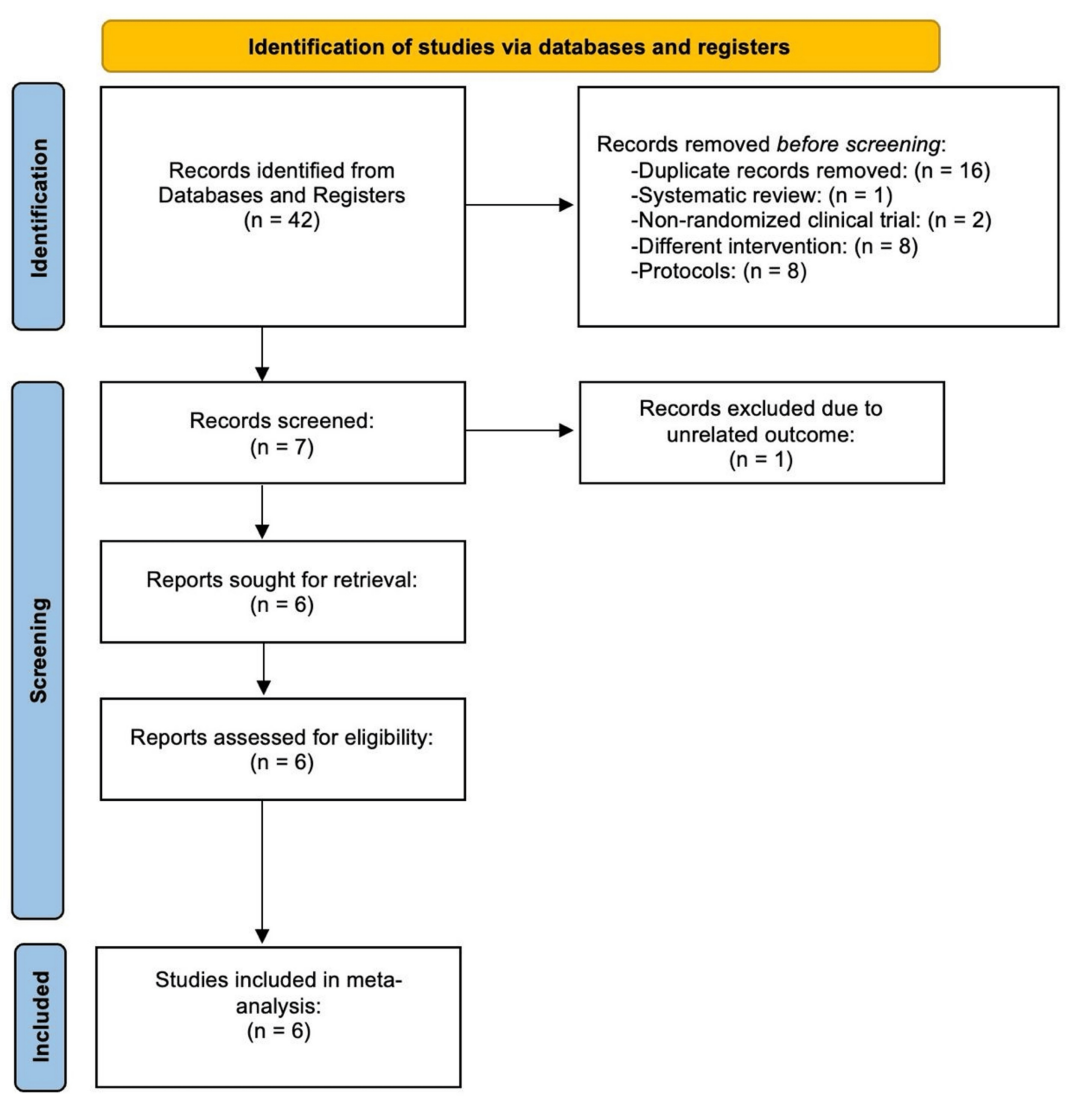
A PRISMA flowchart outlining the study selection process PRISMA: Preferred Reporting Items for Systematic Reviews and Meta-Analyses

**Figure 2:**
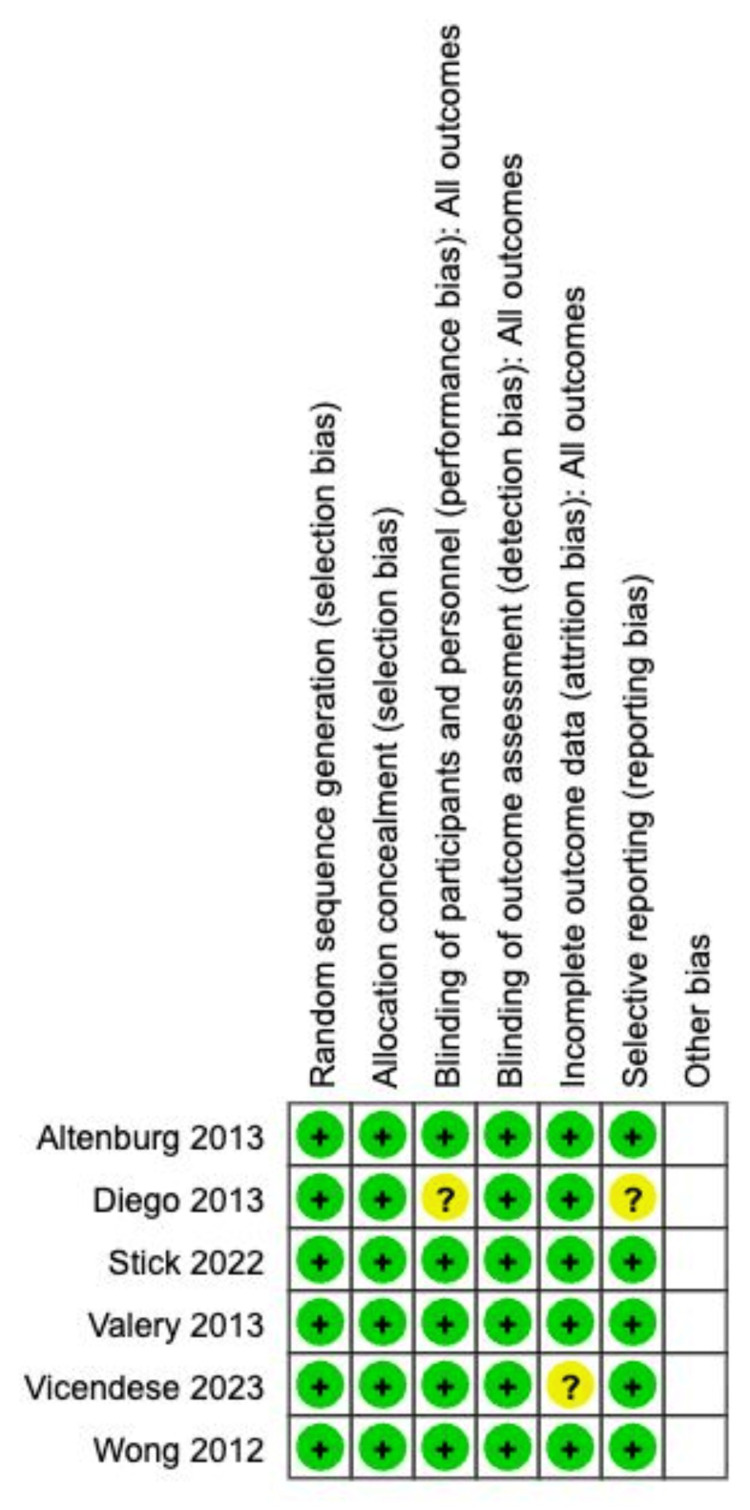
Summary of risk of bias Studies: Altenburg et al. (2013) [7]; Diego et.al. (2013) [9]; Stick et.al. (2022) [5]; Valery et.al. (2013) [10]; Vicendese et.al. (2023) [6]; Wong et.al (2012) [8]

The systematic search and screening process, detailed in the PRISMA flow diagram (Figure 1), resulted in the inclusion of six RCTs for qualitative and quantitative synthesis [4,5,7-10]. These studies provided data for a total of 562 patients (288 in the azithromycin group and 274 in the placebo group). The characteristics of the included studies are presented in Table 1. All studies compared a long-term regimen of azithromycin (ranging from three to 24 months, typically three times weekly) with placebo. Assessment of the risk of bias using the Cochrane RoB tool showed that the included studies were generally of high quality (Figure 2). We deemed all six studies to have a low risk of bias in random sequence generation, allocation concealment, and blinding of participants/personnel. The work by Diego et al. had unclear blinding of personnel [9], and the study by Vicandese et al. raised concerns about incomplete outcome data [4]. Selective reporting bias was low across all trials.

**Table 1:** Characteristics of the studies included FEV1: forced expiratory volume in 1 second; SGRQ: St George’s Respiratory Questionnaire; EBC: exhaled breath condensate; mg: milligram; kg: kilogram; OD: once a day Studies: Wong et.al (2012) [8], Altenburg et al. (2013) [7], Valery et.al. (2013) [10], Diego et.al. (2013) [9], Stick et.al. (2022) [5], Vicendese et.al. (2023) [6]

Author (Year)	Population	Number of Participants	Intervention	Control	Outcomes
Wong et al. (2012) [8]	Adults with non-cystic fibrosis bronchiectasis	141	Azithromycin (500mg tab OD three times a week for six months)	Placebo	Rate of event-based exacerbations in the first six months, FEV1 before bronchodilation, and SGRQ total score at the end of the treatment period
Altenburg et al. (2013) [7]	Adults with non-cystic fibrosis bronchiectasis	83	Oral azithromycin (250mg tab OD for 12 months)	Placebo	Number of infectious exacerbations during the 52-week treatment period.
Valery et al. (2013) [10]	Children aged one to eight years with either bronchiectasis or chronic suppurative lung disease	89	Azithromycin (30 mg/kg) once a week for up to 24 months	Placebo	Exacerbation (respiratory episodes treated with antibiotics) rate.
Diego et al. (2013) [9]	Adult patients with a documented diagnosis of bronchiectasis based on clinical data and a high-resolution computed tomography lung scan	30	Azithromycin 250 mg three times per week for three months	Placebo	Changes in nitric oxide, 8-isoprostane, pH, nitrites, and nitrates in EBC, pulmonary exacerbations
Stick et al. (2022) [5]	Infants (aged three to six months) diagnosed with cystic fibrosis	130	Azithromycin (10 mg/kg bodyweight orally three times per week) until age 36 weeks	Placebo	Proportion of children with radiologically defined bronchiectasis, and the percentage of total lung volume affected by disease, Pulmonary exacerbations, and inflammatory markers
Vicendese et al. (2023) [6]	indigenous children with bronchiectasis unrelated to cystic fibrosis.	89	Azithromycin (30 mg/kg once weekly) for 12 to 24 months	Placebo	Respiratory exacerbation

**Figure 3:**
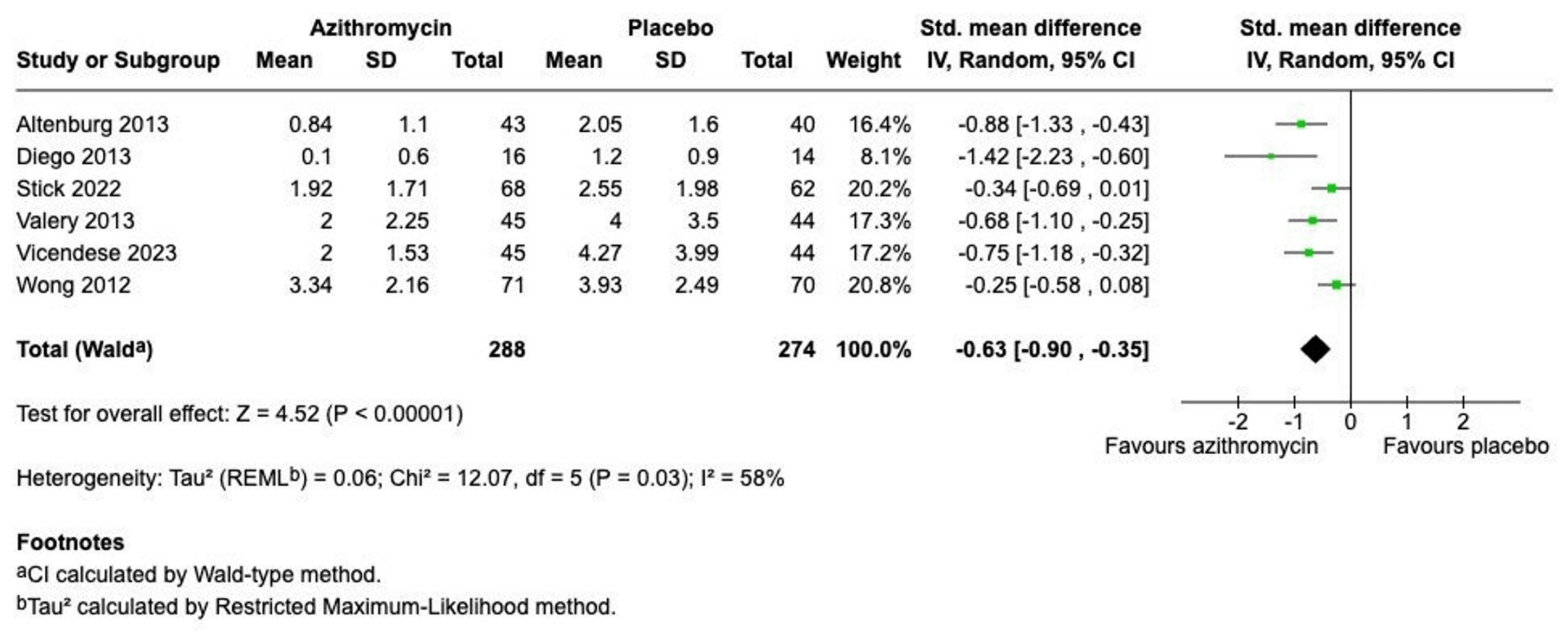
Forest plot of the number of pulmonary exacerbations comparing azithromycin versus placebo Studies: Altenburg et al. (2013) [7]; Diego et.al. (2013) [9]; Stick et.al. (2022) [5]; Valery et.al. (2013) [10]; Vicendese et.al. (2023) [6]; Wong et.al (2012) [8]

**Figure 4:**
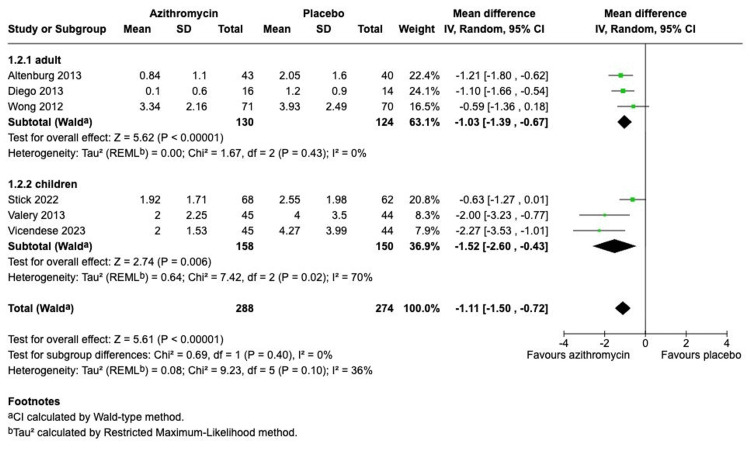
Subgroup analysis of the number of pulmonary exacerbations stratified by population Studies: Altenburg et al. (2013) [7]; Diego et.al. (2013) [9]; Stick et.al. (2022) [5]; Valery et.al. (2013) [10]; Vicendese et.al. (2023) [6]; Wong et.al (2012) [8]

The meta-analysis of the primary outcome, the number of pulmonary exacerbations per patient, demonstrated a statistically significant reduction in the rate of pulmonary exacerbations in the azithromycin group compared to placebo (Figure 3). The pooled analysis, performed using a random-effects model, yielded an SMD of -0.63 (95% CI, -0.90 to -0.35). The test for overall effect was highly significant (Z = 4.52, P < 0.00001), with moderate heterogeneity observed across the trials (I^2^ = 58%). A pre-specified subgroup analysis stratified by patient population (adult vs. children) showed a similar direction of effect in both groups (Figure 4). The adult subgroup (N=254) reported an SMD of -1.03 (95% CI, -1.39 to -0.67), with low heterogeneity (I^2^ = 0%). The children subgroup (N=308) reported a larger effect size, SMD -1.52 (95% CI, -2.60 to -0.43), but with high heterogeneity (I^2^ = 70%). Notably, the test for subgroup differences showed no statistically significant difference in the magnitude of the treatment effect between adults and children (chi-square = 0.69, df = 1, p = 0.43).

**Figure 5:**
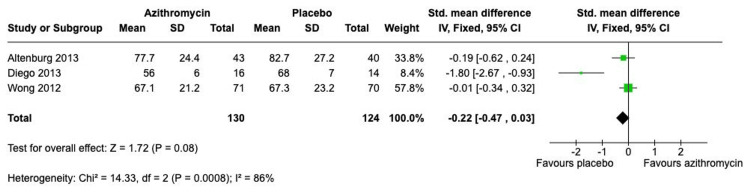
Forest plot for predicted FEV1 FEV1: forced expiratory volume in 1 second Studies: Altenburg et al. (2013) [7]; Diego et.al. (2013) [9]; Wong et.al (2012) [8]

**Figure 6:**
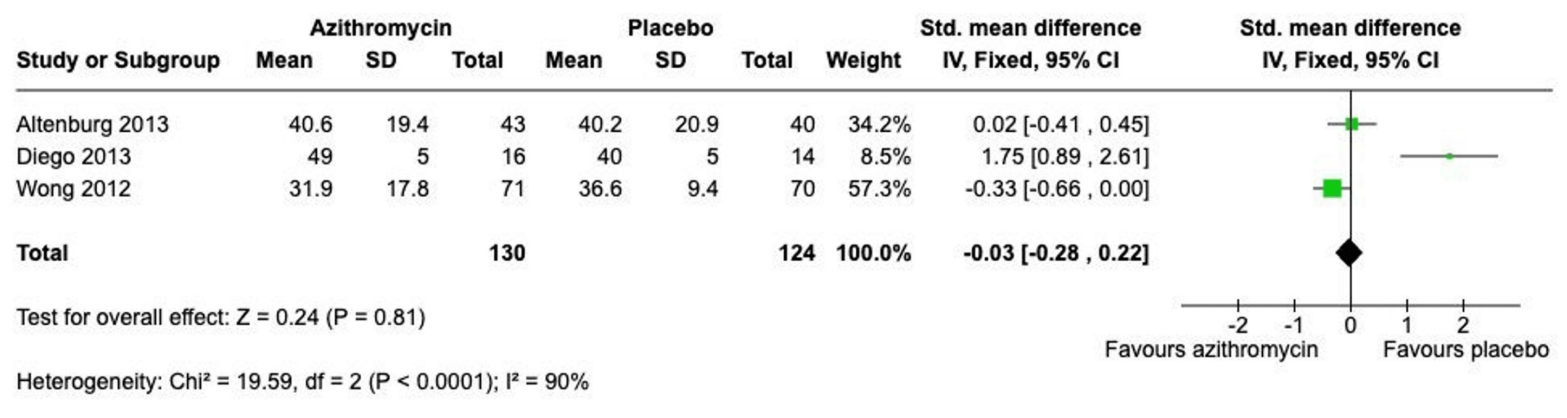
Forest plot for St. George’s Respiratory Questionnaire Score (SGRQ) Studies: Altenburg et al. (2013) [7]; Diego et.al. (2013) [9]; Wong et.al (2012) [8]

**Figure 7:**
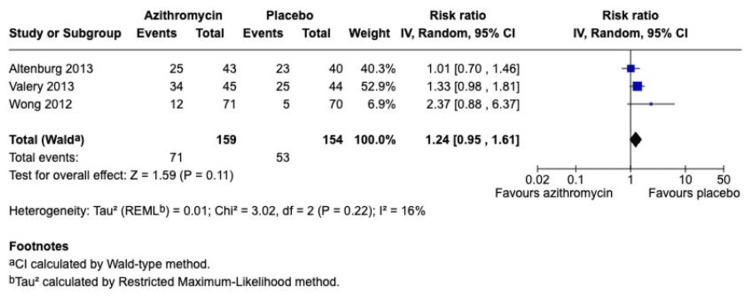
Forest plot for no adverse events Studies: Altenburg et al. (2013) [7]; Valery et.al. (2013) [10]; Wong et.al (2012) [8]

Analysis of secondary outcomes suggested that the benefit of azithromycin is primarily confined to exacerbation prevention, with little impact on lung function or quality of life. The pooled analysis for FEV1 percent predicted, including three adult studies (Figure 5), showed no statistically significant difference between the intervention and control groups (SMD -0.22; 95% CI, -0.47 to 0.03; p = 0.08), but with very high heterogeneity (I^2^ = 86%). Similarly, the meta-analysis for the SGRQ total score showed no significant overall effect (SMD -0.03; 95% CI, -0.28 to 0.22; p = 0.81), confounded by extremely high heterogeneity (I^2^ = 90%) (Figure 6). Regarding safety, the analysis of the overall adverse events, including three studies (Figure 7), showed no statistically significant difference in the risk of experiencing adverse events between the two groups, with an overall RR of 1.24 (95% CI, 0.95 to 1.61; p = 0.11).

**Figure 8:**
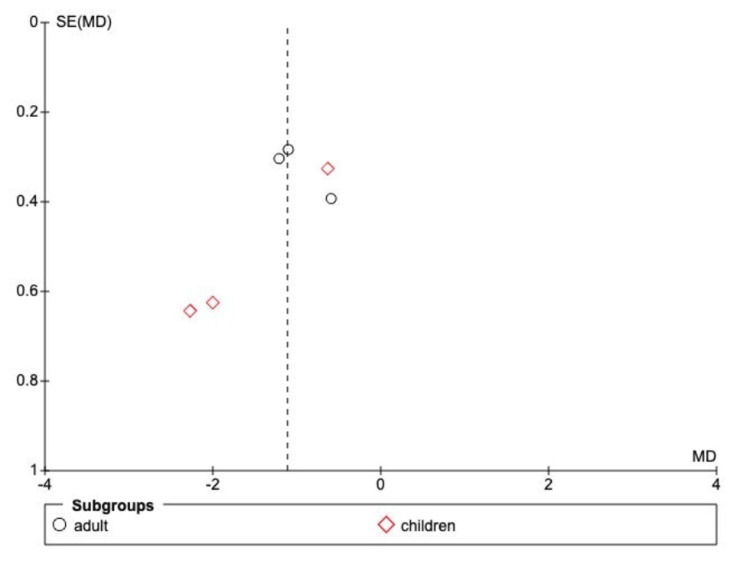
Funnel plot for publication bias visual assessment

Visual inspection of the funnel plot (Figure 8) revealed noticeable asymmetry. The clustering of trials in the area indicating a strong treatment effect, combined with the absence of smaller trials showing null or negative results, suggests the presence of small-study effects or potential selective publication bias. Given the small number of included trials (N=6), these findings should be interpreted with caution.

Discussion

This updated systematic review and meta-analysis of six high-quality RCTs confirms the significant clinical efficacy of long-term prophylactic azithromycin in reducing the frequency of pulmonary exacerbations in patients with bronchiectasis. A critical distinction must be made between statistical and clinical significance. Statistically, the results are highly robust (P < 0.00001). Clinically, the SMD of -0.63 represents a moderate-to-large effect size. Our findings extend previous meta-analyses by Lee et al. [6] and Li et al. [11], providing more precise estimates by incorporating high-impact recent trials [4, 5]. 

The subgroup analysis by population is the most critical finding of the systematic review and meta-analysis, where the treatment effect is consistent and statistically equivalent in adults (SMD -1.03) and children (SMD -1.52); the test for subgroup differences was non-statistically significant (p = 0.43). It provides essential evidence to guide pediatric practice, which has been limited until now by a lack of high-certainty data [6]. However, the safety analysis (Figure 7) is reassuring, as there was no statistically significant increase in overall adverse events between the azithromycin and placebo groups (p = 0.11). The pooled analysis may obscure specific adverse events reported in individual studies, such as gastrointestinal upset, and the trials did not address the long-term concern about macrolide resistance [12].

While the primary outcome clearly favors azithromycin, the secondary outcome analysis provides critical clinical context. Azithromycin conferred no statistically significant benefit on lung function, as assessed by FEV1 (p = 0.08), or on health-related quality of life, as assessed by the SGRQ score (p = 0.81). This is an essential distinction for counseling patients about what to expect from their treatment. 

An important aspect of these findings is the underlying physiological mechanism. The efficacy of azithromycin is likely attributed to its pleiotropic effects, targeting the "vicious cycle" of bronchiectasis, a self-perpetuating loop of impaired mucociliary clearance, bacterial colonization, and neutrophilic inflammation [1, 2]. Beyond its direct antibacterial activity against common pathogens like Haemophilus influenzae, azithromycin exerts potent immunomodulatory effects, including the inhibition of pro-inflammatory cytokines like IL-8 and a reduction in mucus hypersecretion [9, 12]. This, in turn, may well explain the action of the drug in preventing acute infectious triggers in the absence of significant improvement in baseline FEV1 or quality of life, as represented by the SGRQ. Thus, azithromycin acts more as a prophylactic agent rather than a disease-modifying agent to reverse established lung damage or substantially improve baseline well-being.

Sources of Heterogeneity

Overall heterogeneity was moderate (I² = 58%), while the pediatric subgroup showed high heterogeneity (I² = 70%). This is likely due to the natural variability in clinical and methodological characteristics. Clinically, patients ranged from infants with cystic fibrosis [5] and indigenous patients [4] to the elderly, representing different levels of underlying inflammation and microbial infection. Methodologically, variability arose from different dosing regimens and study durations (three to 24 months). However, despite this statistical heterogeneity, there was absolute consistency in the direction of effect across all studies in favor of azithromycin. The high heterogeneity in the FEV1 (I² = 86%) and SGRQ (I² = 90%) analyses can also be attributed to the methodological differences, variable baseline disease severity, and different tools used to measure these outcomes.

Limitations

The main limitation remains the absence of long-term follow-up data (>12 months), which are essential for a complete assessment of the cumulative effect of macrolide use on the prevalence of macrolide-resistant organisms and long-term cardiac events. Further, high heterogeneity across most secondary outcomes and the visual asymmetry in the funnel plot (Figure 5) suggest publication bias, which should be interpreted with caution. The risk of bias assessment (Figure 2) was low; however, given the heterogeneity in dosing, frequency, and duration across the six studies (I² = 58% for the primary outcome), it is difficult to establish a single regimen as superior. 

Recommendations

These findings strongly support the current use of long-term azithromycin as an effective strategy in reducing exacerbations in bronchiectasis, independent of age. Clinical guidelines should embed this robust evidence, especially for pediatric recommendations. Future research in this area should prioritize (1) long-term (multi-year) observational studies or extended RCTs to quantify the risk of macrolide resistance development; (2) studies aimed at identifying biomarkers (e.g., microbiological profile, sputum inflammatory markers) that predict treatment response; and (3) investigations into alternative, potentially non-antibiotic, anti-inflammatory agents to address the limitations of long-term macrolide use.

## Conclusions

Long-term prophylactic azithromycin significantly decreases the frequency of pulmonary exacerbations in patients with bronchiectasis. The consistent benefit observed in both adult and pediatric patient populations enables a therapy recommendation that is generalizable across many patients with bronchiectasis. Although it fails to show significant improvement in baseline lung functions and quality-of-life measures, its effectiveness in lowering the incidence of acute exacerbations gives it an essential role in treatment regimens in patients who experience recurring episodes. It also has to be used in a balanced manner, considering that despite its benefits, there remain issues concerning its effects on antimicrobial resistance and long-term cardiac effects.
